# *SLC5A2* mutations, including two novel mutations, responsible for renal glucosuria in Chinese families

**DOI:** 10.1186/s12882-020-01725-9

**Published:** 2020-02-28

**Authors:** Lei Yu, Meng Wu, Ping Hou, Hong Zhang

**Affiliations:** 1 Renal Division, Inner Mongolia People’s Hospital, Hohhot, Inner Mongolia 010017 People’s Republic of China; 2 Department of Nephrology, Longyan First Hospital Affiliated to Fujian Medical University, Longyan, Fujian 364000 People’s Republic of China; 3 Renal Division, Peking University First Hospital; Peking University Institute of Nephrology; Key Laboratory of Renal Disease, Ministry of Health of China, Beijing, 100034 People’s Republic of China

**Keywords:** Diabetes, Familial renal glucosuria, Mutation, Permanent growing lymphoblastoid cell line, *SLC5A2*, SGLT2

## Abstract

**Background:**

Familial renal glucosuria (FRG) is characterized by persistent glucosuria without other impairments of tubular function in the presence of normal serum glucose. SGLT2, which is almost exclusively expressed in the kidney, accounts for most of the glucose reabsorption. Recently, some studies have confirmed that *SLC5A2* mutations are responsible for the pathogenesis of familial renal glucosuria, but FRG cases are still rare. Furthermore, there are a few reports about splice-site mutations in previous studies, but the effect of these variants at the mRNA level has hardly been verified.

**Methods:**

Ten patients were recruited in our renal division because of persistent glucosuria, and clinical data of the patients and their family members were recorded as much as possible. The entire coding region and adjacent intronic segments of *SLC5A2* were sequenced in FRG patients and their relatives. Permanent growing lymphoblastoid cell lines from FRG patients were established to better preserve genetic information.

**Results:**

A total of nine different mutations were identified: IVS1-16C > A, c.305C > T/p.(A102V), c.395G > A/p.(R132H), c.736C > T/p.(P246S), c.886(−10_-31)delGCAAGCGGGCAGCTGAACGCCC, c.1152_1163delGGTCATGCTGGC/p.(Val385_Ala388del), c.1222G > T/p.(D408Y), c.1496G > A/p.(R499H) and c.1540C > T/p.(P514S); two novel mutations in *SLC5A2*, c.1222G > T/p.(D408Y) and c.1496G > A/p.(R499H), were identified in the Chinese FRG pedigrees. Ten individuals with heterozygous or compound heterozygous variants had glucosuria in the range of 3.1 to 37.6 g/d.

**Conclusion:**

We screened ten additional Chinese FRG pedigrees for mutations in the *SLC5A2* gene and found nine mutations, including two novel mutations. Most variants were private, but IVS1-16C > A and c.886(−10_-31) del may be high frequency splice-site mutations that could be preferentially screened when variants cannot be found in the *SLC5A2* exon. Furthermore, we successfully established a permanent growing lymphoblastoid cell line from patients with FRG, which could facilitate further studies of the *SLC5A2* gene. The current study provides a valuable clue for further research on the molecular mechanism of SGLT2.

## Background

Familial renal glycosuria (FRG) is characterized by persistent glycosuria with normal blood sugar concentrations and without any other impairment of tubular function [1]. The main reabsorptive mechanism for D-glucose in the kidney involves a lower affinity, high capacity Na(+)/glucose cotransporter 2 (SGLT2), which is located in the S1 segment of the early proximal convoluted tubule, and a Na(+) and glucose coupling ratio of 1:1 [2]. The *SLC5A2* gene was mapped to 16p11.2 [3]. Recently, some published studies have confirmed that *SLC5A2* mutations are responsible for FRG patients [4–16]. In some of these studies, FRG was considered an autosomal recessive disorder [7–11]. In others, it was considered a codominant trait with variable penetrance [5, 6]. In our previous studies, the inheritance of renal glucosuria was best described as codominant with a variable penetrance in relation to the compensatory capacity of wild-type [12, 14, 15]. In long-term follow-up studies, the outcome of FRG patients is very good [5, 17]. SGLT2 inhibitors are designed to improve the condition of diabetes without increasing the risk of weight gain or hypoglycemia. SGLT2 has been the subject of particular attention in the search for potential new drugs for the treatment of diabetes [18]. Here, we describe ten patients with glucosuria of variable severity and nine *SLC5A2* mutations. Furthermore, in previous reports, the effect of splice-site variants was rarely verified. We established a permanent growing lymphoblastoid cell line to verify the effect of splice-site variants from previous studies [12].

## Methods

Patients with FRG were diagnosed by persistent glycosuria in the presence of a normal serum glucose concentration and no other impairments of tubular function or any other type of renal disease. Ten unrelated FRG patients and their families were investigated as much as possible. The age, sex, serum creatinine, urine protein excretion, glucosuria excretion and other clinical manifestations in all patients were recorded. Fifty-five healthy Chinese individuals were included as controls in our study.

Genomic DNA was extracted by a salting out procedure from peripheral white blood cells from whole blood samples [19]. The products of polymerase chain reaction (PCR) were sequenced, and the genomic DNA reference sequence of *SLC5A2* (NG_012892.1, Gene ID: 6524) and protein reference sequence of SGLT2 (NP_003032.1) were acquired from the Entrez gene and protein databases, respectively. In the analysis of variants, the entire coding region and adjacent intronic segments of *SLC5A2* were sequenced in family members as much as possible, and the variants were confirmed by bidirectional sequencing. The set of primers used was previously reported [11]. We established a permanent growing lymphoblastoid cell line from patients with FRG as previously reported [12, 20].

A total of 110 control chromosomes were tested by sequencing or polymerase chain reaction-restriction fragment length polymorphism (PCR–RFLP) to rule out common polymorphisms. Furthermore, three databases, including the Exome Aggregation Consortium (ExAC, http://exac.broadinstitute.org/), GnomAD v3 and GnomAD v2.1.1 (http://gnomad.broadinstitute.org), were used to further eliminate polymorphisms.

Amino acid substitutions were evaluated using the in silico prediction programs SIFT and PolyPhen-2. In addition, a comparative analysis of multiple amino acid sequences of SGLT2 was performed in different species by multiple sequence alignments of DNAMAN Version 6. The aligned reference sequences of *Homo sapiens* (NP_003032.1), *Pan troglodytes* (XP_009428973.2), *Macaca mulatta* (XP_001113206.3), *Bos taurus* (NP_976236.1), *Rattus norvegicus* (NP_072112.2), *Mus musculus* (NP_573517.1), *Danio rerio* (NP_998091.1) and *Xenopus tropicalis* (XP_002940641.2) were used to evaluate the evolutionary conservation.

## Results

**Fig. 1:**
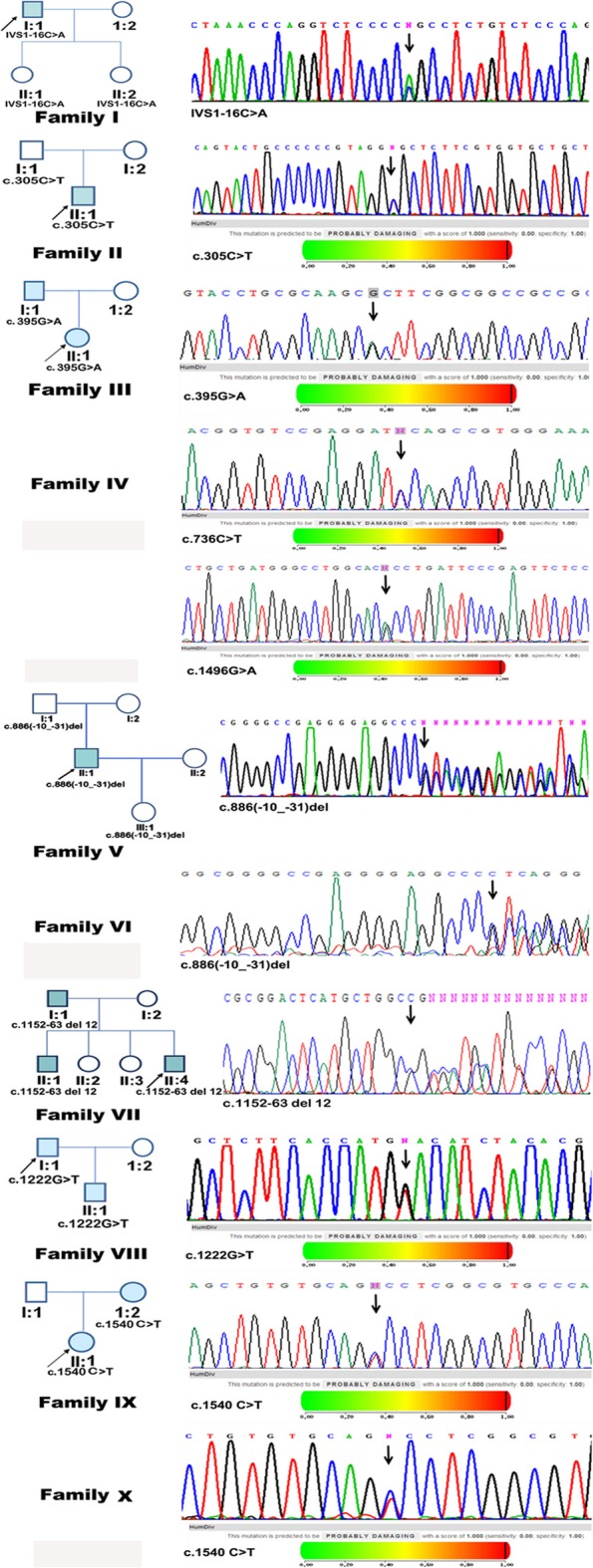
Ten familial renal glucosuria pedigrees carry *SLC5A2* variants. A total of nine different mutations were identified: IVS1-16C > A, c.305C > T/p.(A102V), c.395G > A/p.(R132H), c.736C > T/p.(P246S), c.886(−10_-31)delGCAAGCGGGCAGCTGAACGCCC, c.1152_1163delGGTCATGCTGGC/p.(Val385_Ala388del), c.1222G > T/p.(D408Y), c.1496G > A/p.(R499H) and c.1540C > T/p.(P514S); two novel mutations in *SLC5A2*, c.1222G > T/p.(D408Y) and c.1496G > A/p.(R499H), were identified in the Chinese FRG pedigrees. Ten individuals were heterozygous or compound heterozygous for an SGLT2 mutation, resulting in glucosuria. The missense variants were predicted to be possibly damaging by PolyPhen-2

All ten patients met the diagnostic criteria of FRG. These patients and their families did not have any other tubular dysfunctions or any other type of renal disease. A total of nine different mutations were identified: IVS1-16C > A, c.305C > T/p.(A102V), c.395G > A/p.(R132H), c.736C > T/p.(P246S), c.886(−10_-31) delGCAAGCGGGCAGCTGAACGCCC, c.1152_1163delGGTCATGCTGGC/p.(Val385_Ala388del), c.1222G > T/p.(D408Y), c.1496G > A/p.(R499H) and c.1540C > T/p.(P514S); two novel mutations in *SLC5A2*, c.1222G > T/p.(D408Y) and c.1496G > A/p.(R499H), were identified in the Chinese FRG pedigrees (Fig. 1). By PCR–RFLP testing or sequencing, these variants were not found in one hundred and ten chromosomes derived from the fifty-five healthy unrelated individuals (Table 1). Because allele frequencies for the observed variants in the Chinese population are still unknown, extremely low allele frequencies of these variants were alternatively obtained in East Asian patients from the ExAC and gnomAD databases (Table 2).

**Table 1:** Mutations and glucose excretion in the patients and their relativ**es**

Family members (age^a^)	Glucose excretion^b^	Allele 1	Allele 2	Confirmation^c^
Family I				
I:1 (62)	**9.6 g/24 h**	**IVS1-16C > A**	**WT**	**AciI,Sequencing**
I:2 (60)	**–**	**WT**	**WT**	**AciI,Sequencing**
II:1 (36)	**–**	**IVS1-16C > A**	**WT**	**AciI,Sequencing**
II:2(34)	**–**	**IVS1-16C > A**	**WT**	**AciI,Sequencing**
Family II				
I:1 (42)	**–**	**c.305C > T/p.(A102V)**	**WT**	**HaeII**
I:2 (39)	**–**	**WT**	**WT**	**HaeII**
II:1 (20)	**19.6 g/24 h**	**c.305C > T/p.(A102V)**	**WT**	**HaeII**
Family III				
I:1 (50)	**1+**	**c.395G > A/p.(R132H)**	**WT**	**HaeII**
I:2 (48)	–	**WT**	**WT**	**HaeII**
II:1 (23)	**7.9 g/24 h**	**c.395G > A/p.(R132H)**	**WT**	**HaeII**
Family IV				
II:4 (47)	**37.6 g/24 h**	**c.736C > T/p. (P246S)**	**c.1496G > A/p.(R499H)**	**BamH I** **StyI, Sequencing**
Family V				
I:1 (63)	**–**	**c.886(−10_-31)del**	**WT**	**10% 29:1 PAGE Gel**
I:2 (61)	**–**	**WT**	**WT**	**10% 29:1 PAGE Gel**
II:1 (39)	**18.7 g/24 h**	**c.886(−10_-31)del**	**WT**	**10% 29:1 PAGE Gel**
II:2 (38)	**–**	**WT**	**WT**	**10% 29:1 PAGE Gel**
III:1 (12)	**–**	**c.886(−10_-31)del**	**WT**	**10% 29:1 PAGE Gel**
Family VI				
I:1 (31)	**8.3 g/24 h**	**c.886(−10_-31)del**	**WT**	**10% 29:1 PAGE Gel**
Family VII				
I:1 (66)	**2+**	**c.1152–63 del/** **p.(Val385_Ala388del)**	**WT**	**Sequencing**
I:2 (64)	**–**	**WT**	**WT**	**Sequencing**
II:1 (40)	**1+**	**c.1152–63 del/** **p.(Val385_Ala388del)**	**WT**	**Sequencing**
II:2 (38)	**–**	**WT**	**WT**	**Sequencing**
II:3 (36)	**–**	**WT**	**WT**	**Sequencing**
II:4 (32)	**3.1 g/24 h**	**c.1152–63 del/** **p.(Val385_Ala388del)**	**WT**	**Sequencing**
Family VIII				
I:1 (47)	**3.6 g/24 h**	**c.1222G > T/p.(D408Y)**	**WT**	**Sty I,Sequencing**
I:2 (45)	**–**	**WT**	**WT**	**Sty I,Sequencing**
II:1 (22)	**1+**	**c.1222G > T/p.(D408Y)**	**WT**	**Sty I,Sequencing**
Family IX				
I:1 (61)	**–**	**WT**	**WT**	**Sequencing**
I:2 (61)	**2+**	**c.1540 C > T/P.(P514S)**	**WT**	**Sequencing**
II:1 (37)	**7.1 g/24 h**	**c.1540 C > T/P.(P514S)**	**WT**	**Sequencing**
Family X				
I:1 (50)	**11.8 g/24 h**	**c.1540 C > T/p.(P514S)**	**WT**	**Sequencing**

^a^In years, at time of evaluation

^b^Quantitative (g/24 h) or qualitative test for glucose in urine. The code “**-**” means not present in qualitative test

^c^Loss of a restriction site for the indicated enzyme in the presence of the mutation. The identified mutations were not detected in110 chromosomes derived from 55 healthy, unrelated individuals, indicating that these mutations do not represent common polymorphisms

**Table 2:** Allele frequencies for the variants in the East Asia population

Allele	ExAc Allele Frequency		GnomAD V3 Allele Frequency		GnomAD V2.1 Allele Frequency	
IVS1-16C > A	0.0001172	1/8532	Not found	Not found	0.0001634	3/18356
c.305C > T/p.(A102V)	0	0/8622	0	0/3130	0	0/19944
c.395G > A/p.(R132H)	0	0/8638	0	0/3134	0	0/19944
c.736C > T/p. (P246S)	0.0005845	5/8554	0.0003193	1/3132	0.0004015	8/19926
c.886(−10_-31)del	Not found	Not found	Not found	Not found	0.0001111	2/18002
c.1152-63*del*	0	0/8170	0	0/3134	0	0/19432
c.1222G > T/p.(D408Y)	Not found	Not found	Not found	Not found	Not found	Not found
c.1496G > A/p.(R499H)	0	0/8596	Not found	Not found	0	0/18370
c.1540 C > T/p.(P514S)	0.001508	13/8620	0.001276	4/3134	0.001254	25/19936

“Not found” means not present in database

**Fig. 2:**
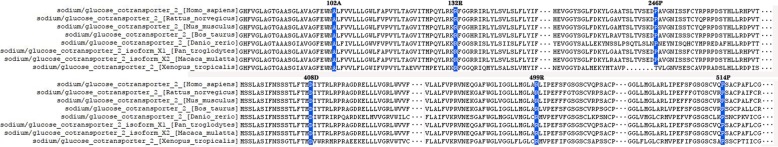
Multiple sequence alignment of the SGLT2 protein from different species. Six conserved amino acids in SGLT2 were identical among different species and are highlighted

The identified missense variants are highly conserved in SGLT2 homologs in multiple species (Fig. 2). By PolyPhen-2 [21], all of these missense variants were predicted to be “probably damaging” (Fig. 1, Table 3). Consistent with PolyPhen-2, five missense variants, c.305C > T/p.(A102V), c.395G > A/p.(R132H), c.1222G > T/p.(D408Y), c.1496G > A/p.(R499H) and c.1540C > T/p.(P514S), were predicted by SIFT to “affect protein function”, but the c.736C > T/p.(P246S) variant was predicted to be “tolerated” by SIFT, which was different from the PolyPhen-2 prediction (Table 3).

**Table 3:** Prediction effect of six missense variants in SLC5A2 gene were performed by PolyPhen-2 and SIFT

Missense variants	PolyPhen-2		SIFT	
	Predicted	Score	Predicted	Score
c.305C > T/p.(A102V)	PROBABLY DAMAGING	1	AFFECT PROTEIN FUNCTION	0.01
c.395G > A/p.(R132H)	PROBABLY DAMAGING	1	AFFECT PROTEIN FUNCTION	0.00
c.736C > T/p. (P246S)	PROBABLY DAMAGING	1	TOLERATED	0.42
c.1222G > T/p.(D408Y)	PROBABLY DAMAGING	1	AFFECT PROTEIN FUNCTION	0.00
c.1496G > A/p.(R499H)	PROBABLY DAMAGING	1	AFFECT PROTEIN FUNCTION	0.00
c.1540 C > T/p.(P514S)	PROBABLY DAMAGING	1	AFFECT PROTEIN FUNCTION	0.03

Glucosuria ranged from 3.1 to 37.6 g/d in ten patients with *SLC5A2* heterozygous or compound heterozygous variants. Some of the family members with heterozygous variants had increased glucose excretion (Table 1). In these families, inheritance of FRG shows characteristics of a codominant trait with variable penetrance. Most variants were private, but the IVS1-16C > A and c.886(−10_-31) del variants were reported in several unrelated pedigrees from different ethnic origins in our and previous studies [12–16, 22, 23].

## Discussion

Glucose, mainly from carbohydrates, is the fuel that provides energy for human activities. The kidneys reabsorb nearly 180 g of glucose filtered daily to keep blood glucose in the normal range. In previous studies, familial renal glycosuria was characterized by persistent glycosuria, and the SGLT2 protein was found to be mainly responsible for the reabsorption of urinary glucose in renal tubules [1, 24, 25]. Therefore, it was speculated that *SLC5A2* gene mutations lead to familial renal glycosuria. The first report of an *SLC5A2* mutation in FRG was presented in 2000 [26]. Recently, a series of studies have confirmed that *SLC5A2* mutations are indeed responsible for FRG [4–16, 22, 23]. In our previous and current studies, fourteen novel variants in *SLC5A2* were identified in twenty-two Chinese renal glucosuria families and confirm previous observations that most variants were private mutations. With an increasing number of FRG patients being found, some variants, such as 294C > A, IVS1-16C > A, c.886(−10_-31) del, and c.1540G > T, did not occur rarely in our and previous studies [12–16, 22, 23]. However, these variants are difficult to regard as hotspot mutations because they were found in *SLC5A2* with a relatively dispersed distribution. Whereas the IVS1-16C > A and c.886(−10_-31) del variants are reported in several unrelated pedigrees of different ethnic origins, these two splice-site variants might be preferentially screened in FRG patients when the other variants cannot be found in the *SLC5A2* exon. Furthermore, specific novel primers should be developed to check for the presence of the observed splice site variants in genomic DNA in future studies.

In the current study, a total of nine different mutations in *SLC5A2* were identified in the Chinese FRG pedigrees. None of these variants were found in one hundred and ten chromosomes from healthy unrelated individuals. In addition, the allele frequencies for these variants were extremely low in East Asian populations. For the identified missense variants, five variants, c.305C > T/p.(A102V), c.395G > A/p.(R132H), c.1222G > T/p.(D408Y), c.1496G > A/p.(R499H) and c.1540C > T/p.(P514S), were highly conserved in SGLT2 homologs in multiple species and were predicted to be “probably damaging” or to “affect protein function” by PolyPhen-2 and SIFT. Only the variant of c.736C > T/p. (P246S) was an exception, and was predicted by SIFT to be “tolerated”, but this variant was reported in previous studies [12, 27] and was confirmed by having a significantly lower glucose transport capacity in cultured cells [12]. Therefore, based on the extremely low allele frequencies of these mutations, highly conservative predictions from biological software and previous studies, it can be safety speculated that these variants are not common polymorphisms and are pathogenic mutations.

In previous studies, many heterozygous individuals presented with mild glucosuria (< 10 g/d), while homozygous or compound heterozygous patients usually present with severe renal glucosuria over 10 g/d [4, 5]. The heterozygosity of *SLC5A2* mutations, no matter what kind of mutation (such as nonsense, splice-site, and missense mutations), can lead to mild glucosuria. Consistent with previous research, six individuals were heterozygous for *SLC5A2* variants resulting in mild glucosuria (< 10 g/d), and one compound heterozygous patient from Family II had severe renal glucosuria (37.6 g/d) in the current study. It is very interesting that three heterozygous patients from Families II, V, and X resulted in severe renal glucosuria (> 10 g/d), and further studies are needed to uncover the related regulatory mechanism.

In the current study, an autosomal codominant trait with variable penetrance inheritance was found in FRG families. In our previous studies, we found that the inheritance of renal glucosuria should be described as codominant with a variable penetrance in relation to the compensatory capacity of wild-type [14, 15]. Different modes of penetrance inheritance may be decided by different sites or other special regulatory mechanisms. Thus, reporting mutations is crucial not only for unraveling critical residues in the protein but also for obtaining useful information to identify potential new targets for the treatment of diabetes.

Renal biopsy is not obligatory for FRG patients; therefore, *SLC5A2* cDNA from the kidney is almost impossible to obtain. Although there are a few reports about splice-site variants [4, 5, 22, 23], the effect of splice-site variants is very difficult to verify in cDNA. We searched through NCBI GEO profiles and found that the SGLT2 protein can be expressed in peripheral white blood cells and lymphocytes. However, due to the limited expression and lifespan of these cells, new blood sampling is necessary via repeatedly drawing blood for reexamination. This might be difficult or even impossible if patients were not available for different reasons. In 1986, a routine method for the establishment of permanent growing lymphoblastoid cell lines was reported [20]. In a previous report, the Epstein–Barr virus genome not only persists as a plasmid with 5–800 copies per cell in most cell lines but also integrates into the host DNA and has been described for a few cell lines [18]. However, there have been no reports on establishing lymphoblastoid cell lines from FRG families in previous studies. We successfully established a permanent growing lymphoblastoid cell line from patients with FRG and successfully verified the effects of splice-site mutations at the cDNA level [12]. Although the integration into the host DNA may affect genetic information, the integration of the Epstein–Barr virus in lymphoblastoid cell lines is nonrandom [28]. The viral integration sites included 1p, 1q, 2q, 3p, 3q, 4q, 5q, 6q, 7p, 7q, 9q, 11p, 14q and 15q. No viral integration occurred in chromosomes 16–22 or the sex chromosomes [28–30]. Because the *SLC5A2* gene was mapped to 16p11.2 and there were no Epstein–Barr virus gene sequences in the cDNA sequencing results, we confirmed that a permanent growing lymphoblastoid cell line from FRG patients was successfully established. In the current study, we found two splice site variants: IVS1-16C > A and c.1152-63del. However, the effect of these two splice-site variants has been verified in previous studies [13, 22]. Therefore, we did not retest the effect of splice-site variants in cDNA in the current study. However, we still believe that the method for establishing permanent growing lymphoblastoid cell lines in patients with FRG is useful to maintain genetic information about *SLC5A2* and more easily verify the effect of splice-site variants in cDNA*.*

In previous studies, the variant frequency of c.886(−10_-31) del in the Chinese population was as high as 32% [22, 23]. Therefore, we rescreened the observed splice site variants in every patient from the twenty-two Chinese renal glucosuria families that were found in our previous and current studies. Finally, except for splice site mutations that were previously found, no additional splice site variants were discovered in these renal glucosuria families.

## Conclusions

In conclusion, we screened ten additional Chinese FRG pedigrees and found nine *SLC5A2* mutations, including two novel mutations. The variants IVS1-16C > A and c.886(−10_-31) del, which had high frequencies, could be preferentially screened in FRG patients when the variants cannot be found in exons. In addition, we established a permanent growing lymphoblastoid cell line from patients with FRG, which could facilitate further studies of the *SLC5A2* gene at the cDNA level. In short, our study provides valuable clues for further studies of the SGLT2 molecular mechanism and potential targets for the further development of anti-diabetes drugs.

## Data Availability

Additional data used/generated that is not present in the manuscript is available from the corresponding author upon reasonable request.

## Abbreviations

EB: Epstein–Barr virus
FRG: Familial renal glucosuria
GLUT: Glucose transporter
PCR: Polymerase chain reaction
PCR–RFLP: Polymerase chain reaction-restriction fragment length polymorphism
SGLT2: Sodium-glucose cotransporter 2
SLC5: Sodium-glucose cotransporter family
*SLC5A2*: Sodium-glucose cotransporter 2 gene
